# The application of predictive value of diabetes autoantibody profile combined with clinical data and routine laboratory indexes in the classification of diabetes mellitus

**DOI:** 10.3389/fendo.2024.1349117

**Published:** 2024-08-22

**Authors:** Jiawen Xian, Rongrong Du, Hui Yuan, Jingyuan Li, Qin Pei, Yongjie Hao, Xi Zeng, Jingying Wang, Ting Ye

**Affiliations:** ^1^ Department of Laboratory Medicine, The Affiliated Hospital of Southwest Medical University, Sichuan, China; ^2^ School of Basic Medical Sciences and School of Stomatology, Mudanjiang Medical University, Heilongjiang, China

**Keywords:** diabetes autoantibody profile, clinical data, routine laboratory indicators, diabetes typing, nomogram

## Abstract

**Objective:**

Currently, distinct use of clinical data, routine laboratory indicators or the detection of diabetic autoantibodies in the diagnosis and management of diabetes mellitus is limited. Hence, this study was aimed to screen the indicators, and to establish and validate a multifactorial logistic regression model nomogram for the non-invasive differential prediction of type 1 diabetes mellitus.

**Methods:**

Clinical data, routine laboratory indicators, and diabetes autoantibody profiles of diabetic patients admitted between September 2018 and December 2022 were retrospectively analyzed. Logistic regression was used to select the independent influencing factors, and a prediction nomogram based on the multiple logistic regression model was constructed using these independent factors. Moreover, the predictive accuracy and clinical application value of the nomogram were evaluated using Receiver Operating Characteristic (ROC) curves, calibration curves, decision curve analysis (DCA), and clinical impact curves (CIC).

**Results:**

A total of 522 diabetic patients were included in this study. These patients were randomized into training and validation sets in a 7:3 ratio. The predictors screened included age, prealbumin (PA), high-density lipoprotein cholesterol (HDL-C), islet cells autoantibodies (ICA), islets antigen 2 autoantibodies (IA-2A), glutamic acid decarboxylase antibody (GADA), and C-peptide levels. Based on these factors, a multivariate model nomogram was constructed, which had an Area Under Curve (AUC) of 0.966 and 0.961 for the training set and validation set, respectively. Subsequently, the calibration curves demonstrated a strong accuracy of the graph; the DCA and CIC results indicated that the graph could be used as a non-invasive valid predictive tool for the differential diagnosis of type 1 diabetes mellitus, clinically.

**Conclusion:**

The established prediction model combining patient’s age, PA, HDL-C, ICA, IA-2A, GADA, and C-peptide can assist in differential diagnosis of type 1 diabetes mellitus and type 2 diabetes mellitus and provides a basis for the clinical as well as therapeutic management of the disease.

## 1 Introduction

Diabetes mellitus (DM) is a common clinical chronic disease with high rate of morbidity, long duration, and lifelong elevation of glucose levels, accompanied by insufficient secretion of insulin by pancreatic islet β-cells or insufficient biological effect of insulin in the peripheral tissues (1). According to the International Diabetes Federation (IDF), by 2040, there will be 642 million diabetics globally, up from the current projection of 460 million (2). Thus, diabetes has now become a serious and urgent public health problem which requires prior attention.

DM can be classified into type 1 diabetes mellitus (T1DM), type 2 diabetes mellitus (T2DM), other special types of diabetes mellitus and gestational diabetes mellitus on the basis of etiological characteristics. The majority of DM cases are classified as T2DM, accounting for 90-95% of the total diabetic patients, and occur as a result of insulin resistance combined with insufficient insulin secretion. T1DM is the second most common, accounting for 5-10% of the diabetic patients (3). T1DM is caused by insulin insufficiency due to pancreatic β-cells dysfunction or autoimmune disruption, and the patient is dependent on insulin for life (4, 5). Approximately, 5-15% of the adults diagnosed with T2DM may actually have T1DM (6). As a result, up to 50% of the actual T1DM cases may be misdiagnosed as T2DM, i.e., the overall number of T1DM cases is considerably underestimated (7). An accurate differential diagnosis is, therefore, essential for the optimal treatment and avoidance of complications.

Distinction between T1DM and T2DM can usually be done according to clinical criteria, primarily based on clinical presentation, age, and body mass index (BMI). However, owing to disease complexity and the diversity of the population, it is difficult to diagnose the phenotype. Regardingly, the laboratory tests can help to differentiate between T1DM and T2DM by using metabolic tests such as C-peptide, insulin, and insulinogen. In a study by Bolinder (8), total insulinogen and intermediate insulinogen degradation products were measured in the subjects, and there was considerable overlap in the levels of total and intermediate insulinogen between T1DM and T2DM patients 3-4 months after the onset of DM. Katz et al. (9) also showed that C-peptide levels in T1DM and T2DM patients were overlapped. Therefore, the use of these laboratory tests in the differential diagnosis of T1DM and T2DM is constrained.

T1DM is caused by autoimmune β-cells destruction, whereas T2DM is caused by insulin resistance, which causes relative β-cells failure, eventually (10). Therefore, if autoantibodies targeting β-cells are detected, this is suggestive of an autoimmune etiology and can help to diagnose autoimmune T1DM (11). There are currently five diabetes autoantibodies that can be used to diagnose T1DM and predict disease progression in non-T1DM patients, which include islet cells autoantibodies (ICA), insulin autoantibodies (IAA), glutamic acid decarboxylase autoantibodies (GADA), islets antigen 2 autoantibodies (IA-2A), and zinc transporter-8 autoantibodies (ZnT8A) (12). Of these, GADA, ICA and IAA are considered to be the three most important antibodies (13). About 70–80% of the individuals with newly diagnosed T1DM have ICA and GADA. 60% have IA-2A and ZnT8A identified, while IAA is infrequent in adults with newly diagnosed T1DM but is present in 50–60% of adolescents with the disease (14). As the duration of diabetes increases, fewer people remain positive for diabetes autoantibodies other than anti-insulin antibodies (14). As in the case of ICA, they are present for a shorter period of time, appearing only in the early stages of T1DM (15). Moreover, the use of insulin is associated with the production of insulin antibodies, and it is impossible to distinguish between insulin antibodies and IAA after 14 days of insulin treatment (16). In addition, overweight or obese adults with a clinical diagnosis of T2DM may also present with positive diabetic autoantibodies (17). In summary, the diabetic autoantibody profile has limitations in differentiating between T1DM and T2DM.

Some studies have shown that cell counts (18–20), liver function (21, 22), blood lipids (23) levels, etc. are linked with the incidence of DM. To investigate the value of diabetic autoantibodies in combination with the clinical data and routine laboratory indicators in the classification of DM, the present study included 522 diabetic patients. The factors of interest including gender, age, autoantibody profile, C-peptide levels, and other relevant variables were recorded. Logistic regression and nomogram analysis were employed to develop predictive models for T1DM, with the goal of improving risk stratification and guiding personalized interventions for individuals at risk of developing diabetes.

## 2 Materials and methods

### 2.1 Study participants

The participants of this study involved 522 diabetic patients admitted from September 2018 to December 2022 at the Affiliated Hospital of Southwest Medical University. All these patients had been clinically diagnosed in advance. There were 89 cases of T1DM, 46 males and 43 females, aged between 4 to 68 years, with a mean age of (28.7 ± 15.06) years; 433 cases of T2DM, 231 males and 202 females, aged between 11 to 88 years, with a mean age of (58.5 ± 14.03) years. There was no statistically significant difference between the two groups in terms of gender (p > 0.05), however, in regards to age, the difference was statistically significant (p < 0.05). The participants were informed about the content and methodology of the study, whereby, they voluntarily participated and cooperated in the study. The study was approved and consented by the Ethics Committee of the hospital.

Inclusion criteria: ① Meet the diagnostic criteria of DM proposed by WHO in 1999; ② Availability of data on autoantibody profile, C-peptide levels, and other relevant predictive factors; ③ Good mental state; ④ Good compliance, can cooperate with the study and examination; ⑤ No contraindication to examination.

Exclusion criteria: ① Other autoimmune diseases; ② Individuals with a history of other types of diabetes (e.g. monogenic diabetes); ③ Hematological diseases; ④ Malignant tumors; ⑤ Acute and chronic systemic infections; ⑥ Lack of essential data for logistic regression and nomogram analysis.

These criteria were implemented to ensure the relevance and accuracy of the predictions made in this clinical prediction study on diabetes.

### 2.2 Diabetes autoantibodies measurements

To measure diabetes autoantibodies, 3 ml blood was collected from the patients in the early morning. The upper layer of serum was centrifuged at 3000 rpm/min for 10 min. Tenfly Blot-C (YHLO Biotech) was used for the detection, and the reagents were the reagents for the instrument (YHLO Biotech). The islets autoantibodies, including GADA, IAA, ICA, IA-2A and ZnT8A, were tested by immunoblotting.

### 2.3 Clinical biochemistry laboratory measurements

For clinical biochemistry laboratory measurements, participants were instructed to fast overnight, 3 ml of fasting venous blood was collected from the patients in the early morning, centrifuged at 3000 rpm/min for 5 min, and then detected by ADVIA 2400 automatic biochemistry analyzer (SIEMENS), and the reagents were matching reagents (SIEMENS). The test items included: alanine aminotransferase (ALT), aspartate aminotransferase (AST), ALT/AST, total protein (TP), prealbumin (PA), albumin (ALB), globulin (GLO), albumin/globulin (A/G), total bilirubin (TBIL), direct bilirubin (DBIL), indirect bilirubin (IBIL), total bile acids (TBA), r- glutamyl transferase (GGT), alkaline phosphatase (ALP), urea, uric acid (UA), creatinine (Crea), retinol-binding protein (RBP), glomerular filtration rate (GFR), total cholesterol (TC), triglycerides (TG), high-density lipoprotein cholesterol (HDL-C), low-density lipoprotein cholesterol (LDL-C), apolipoprotein A1 (APOA1), apolipoprotein B (APOB), glucose (GLU), glycated serum proteins (GSP), potassium ions (K^+^), sodium ions (Na^+^), chloride ions (Cl^-^), calcium ions (Ca^2+^), carbon dioxide (CO_2_), anion gap (AG), and C-peptide.

### 2.4 Complete blood count

For complete blood count, participants were instructed to fast overnight, 2-5 ml of fasting venous blood was collected from patients in the early morning, and tested by BC-6800 automatic blood cell analyzer (Mindray), and the reagents were matching reagents (Mindray). The test items included: white blood cell count (WBC), neutrophil count (NEU), lymphocyte count (LYM), monocyte count (MONO), eosinophil count (EOS), basophil count (BASO), neutrophil ratio (NEU-R), lymphocyte ratio (LYM-R), monocyte ratio (MONO-R), eosinophil ratio (EOS-R), basophil ratio (BASO-R), red blood cell count (RBC), hemoglobin (HGB), hematocrit (HCT), mean corpuscular volume (MCV), mean corpuscular hemoglobin volume (MCH), mean corpuscular hemoglobin concentration (MCHC), standard deviation in red blood cell distribution width (RDW-SD), coefficient of variation of red blood cell distribution width (RDW-CV), platelet count (PLT), mean platelet volume (MPV), platelet compact volume (PCT), platelet volume distribution width (PDW), and platelets larger cell ratio (P-LCR).

The above-mentioned tests were completed by the same group of experienced testing personnel. The test process fully refers to the standard operating procedures to ensure consistency of test results, quality control according to CNAS-CL02: Accreditation Criteria for the Quality and Competence of Medical Laboratories (ISO 15189:2012, Medical laboratories—Requirements for quality and competence, IDT).

### 2.5 Statistical analysis

In this study, the collected data were randomly divided into two groups in the ratio of 7:3 for the training and validation sets. For variables with missing data points, we imputed values using predictive mean matching and logistic regression methods within the multiple imputation framework. Measurements were expressed as mean ± standard deviation (x ± s). Student’s t-test was used to examine the continuous variables, and chi-square test was used to analyze the categorical variables. In the training cohort, the least absolute shrinkage and selection operator (LASSO) logistic regression analysis was used for multivariate analysis to screen the independent risk factors and build a prediction nomogram for the Group. The Area Under Curve (AUC) of the Receiver Operating Characteristic (ROC) curve and the calibration curve were used to evaluate the accuracy of the nomogram; and the clinical benefits of the nomogram were demonstrated using the Decision Curve Analysis (DCA) and the Clinical Impact Curve (CIC). Statistical analyses were performed using SPSS 27.0, SPSSAU, R 4.2.2, along with the use of MSTATA software. Results with a p-value of <0.05 were considered statistically significant.

## 3 Results

### 3.1 The significant differences in age, biochemistry and CBC levels between the T1DM and T2DM groups

Firstly, the patients were divided into T1DM and T2DM groups according to their clinical diagnosis, and t-test was performed to analyze the age, biochemistry, and CBC levels of a total of 58 indicators in the two groups. According to the results, there were significant differences in age, ALT/AST, PA, HDL-C, TBIL, IBIL, ALP, Crea, RBP, GFR, GLU, GSP, WBC, NEU, LYM, MONO, EOS, EOS-R, BASO-R, RBC, RDW-SD, PLT, PCT, Na^+^, CO_2_, AG, and C-peptide between the two groups. The mean values of age, PA, TBIL, IBIL, Crea, RBP, EOS, EOS-R, BASO-R, RDW-SD, Na^+^, CO_2_, and C-peptide were significantly lower in the T1DM group than in the T2DM group. The mean values of ALT/AST, HDL-C, ALP, GFR, GLU, GSP, WBC, NEU, LYM, MONO, RBC, PLT, PCT, PLT, PCT, Na^+^, CO_2_, and AG levels were significantly higher in the T1DM group (p < 0.05). The result of the Student’s t test is presented in the **Table 1**.

**Table 1:** Results of Student’s t test analysis between T1DM and T2DM.

Index	Type (x ± s)		t	*P* value
	T1DM (n = 89)	T2DM (n = 433)		
Age	28.70 ± 15.06	58.50 ± 14.03	-18.023	0.000
ALT/AST	1.30 ± 1.21	1.03 ± 0.56	2.055	0.043
PA	171.01 ± 65.81	224.73 ± 76.71	-6.156	0.000
HDL-C	1.20 ± 0.37	1.11 ± 0.34	2.248	0.026
ALT	29.60 ± 30.12	28.15 ± 26.51	0.459	0.646
AST	28.41 ± 21.10	25.55 ± 21.65	1.137	0.256
TP	70.21 ± 10.04	70.05 ± 7.29	0.142	0.887
ALB	42.38 ± 6.15	42.53 ± 5.19	-0.226	0.821
GLO	27.61 ± 6.09	27.40 ± 4.65	0.308	0.759
A/G	1.60 ± 0.31	1.60 ± 0.32	-0.039	0.969
TBIL	11.97 ± 5.61	13.56 ± 7.17	-1.973	0.049
DBIL	4.12 ± 2.23	4.21 ± 2.43	-0.326	0.745
IBIL	7.84 ± 4.29	9.47 ± 5.80	-2.513	0.012
TBA	4.88 ± 5.11	5.73 ± 6.13	-1.216	0.225
GGT	46.30 ± 106.63	37.01 ± 37.36	0.811	0.419
ALP	119.63 ± 90.22	90.87 ± 44.18	2.935	0.004
Urea	6.25 ± 5.20	7.19 ± 4.45	-1.750	0.081
UA	350.86 ± 164.81	344.25 ± 117.30	0.360	0.720
Crea	60.19 ± 32.07	78.22 ± 54.19	-4.210	0.000
RBP	28.26 ± 13.15	41.39 ± 15.51	-7.451	0.000
GFR	126.33 ± 33.32	91.98 ± 30.20	9.597	0.000
TC	4.63 ± 1.47	4.60 ± 1.25	0.213	0.832
TG	2.39 ± 2.92	2.23 ± 2.01	0.492	0.624
LDL-C	2.66 ± 0.90	2.74 ± 1.07	-0.750	0.455
APOA1	1.32 ± 0.33	1.34 ± 0.30	-0.575	0.566
APOB	0.89 ± 0.28	0.93 ± 0.45	-0.919	0.358
GLU	17.00 ± 9.87	13.34 ± 7.57	3.933	0.000
GSP	3.41 ± 0.97	2.78 ± 0.73	5.812	0.000
WBC	9.40 ± 5.63	7.36 ± 3.36	3.298	0.001
NEU	6.89 ± 5.16	5.12 ± 3.22	3.114	0.002
LYM	1.88 ± 0.88	1.65 ± 0.67	2.302	0.023
MONO	0.51 ± 0.40	0.41 ± 0.19	2.459	0.016
EOS	0.09 ± 0.15	0.14 ± 0.17	-2.524	0.012
BASO	0.03 ± 0.02	0.03 ± 0.02	-0.989	0.323
NEU-R	68.30 ± 15.21	67.20 ± 10.38	0.649	0.518
LYM-R	24.35 ± 13.33	24.40 ± 9.22	-0.030	0.976
MONO-R	5.74 ± 2.65	5.73 ± 1.97	0.015	0.988
EOS-R	1.23 ± 1.88	2.13 ± 2.33	-3.921	0.000
BASO-R	0.37 ± 0.27	0.47 ± 0.30	-2.891	0.004
RBC	4.63 ± 0.65	4.46 ± 0.74	1.993	0.047
HGB	137.78 ± 18.11	133.91 ± 21.74	1.770	0.079
HCT	0.41 ± 0.05	0.41 ± 0.06	0.970	0.332
MCV	89.74 ± 6.70	91.19 ± 7.25	-1.741	0.082
MCH	30.02 ± 2.22	30.16 ± 2.54	-0.479	0.632
MCHC	331.25 ± 33.56	329.68 ± 14.97	0.692	0.489
RDW-SD	40.89 ± 4.67	42.13 ± 3.95	-2.623	0.009
RDW-CV	13.08 ± 1.03	13.22 ± 1.13	-1.056	0.292
PLT	239.13 ± 72.52	213.93 ± 73.88	2.941	0.003
MPV	10.78 ± 1.44	10.69 ± 1.54	0.480	0.632
PCT	0.25 ± 0.07	0.23 ± 0.08	3.030	0.003
PDW	15.82 ± 1.84	15.99 ± 1.62	-0.847	0.397
P-LCR	32.06 ± 10.25	31.43 ± 10.67	0.513	0.608
K^+^	4.27 ± 0.68	4.30 ± 0.53	-0.547	0.584
Na^+^	138.78 ± 4.67	140.13 ± 4.13	-2.740	0.006
Cl^-^	105.34 ± 5.69	106.04 ± 7.19	-0.857	0.392
Ca^2+^	2.37 ± 0.19	2.36 ± 0.17	0.192	0.848
CO_2_	21.22 ± 8.14	25.08 ± 3.89	-4.377	0.000
AG	12.39 ± 6.02	9.39 ± 4.52	4.395	0.000
C-peptide	1.12±0.85	2.08±1.48	-8.335	0.000

ALT/AST, alanine aminotransferase/aspartate aminotransferase; PA, prealbumin; HDL-C, high-density lipoprotein cholesterol; ALT, alanine aminotransferase; AST, aspartate aminotransferase; TP, total protein; ALB, albumin; GLO, globulin; A/G, albumin/ globulin; TBIL, total bilirubin; DBIL, direct bilirubin; IBIL, indirect bilirubin; TBA, total bile acids; GGT, r-glutamyl transferase; ALP, alkaline phosphatase; UA, uric acid; Crea, creatinine; RBP, retinol-binding protein; GFR, glomerular filtration rate; TC, total cholesterol; TG, triglycerides; LDL-C :low-density lipoprotein cholesterol; APOA1, apolipoprotein A1; APOB, apolipoprotein B; GLU, glucose; GSP, glycated serum proteins; WBC, white blood cell count; NEU, neutrophil count; LYM, lymphocyte count; MONO, monocyte count; EOS, eosinophil count; BASO, basophil count; NEU-R, neutrophil ratio; LYM-R, lymphocyte ratio; MONO-R, monocyte ratio; EOS-R, eosinophil ratio; BASO-R, basophil ratio; RBC, red blood cell count; HGB, haemoglobin; HCT, hematocrit; MCV, mean corpuscular volume; MCH, mean corpuscular haemoglobin volume; MCHC, mean corpuscular haemoglobin concentration; RDW-SD, standard deviation in red blood cell distribution width; RDW-CV, coefficient of variation of red blood cell distribution width; PLT, platelet count; MPV, mean platelet volume; PCT, platelet compact volume; PDW, platelet distribution width; P-LCR, platelets larger cell ratio; K^+^, potassium ions; Na^+^, sodium ions; Cl^-^, chloride ions; Ca^2+^, calcium ions; CO_2:_ carbon dioxide; AG, anion gap.

### 3.2 The regression analysis of age, biochemistry, CBC levels and diabetes typing

Further, logistic regression analysis using the typology of diabetic patients included in this study as the dependent variable (TIDM = 0, T2DM = 1) and the above indicators as independent variables showed that age, ALT/AST, PA, HDL-C, EOS, EOS-R, and C-peptide levels were the factors influencing diabetes typing, and age, PA, EOS, EOS-R, and C-peptide levels were negatively correlated with T1DM typing and positively correlated with T2DM typing (p < 0 05). Moreover, ALT/AST and HDL-C levels were positively correlated with T1DM typing and negatively correlated with T2DM typing (p < 0. 05) (**Table 2**). The variance inflation factor (VIF) of EOS and ESO-R is higher than 5 (**Table 2**), so these two variables are screened out in consideration of the possibility of strong multicollinearity.

**Table 2:** The regression analysis of biochemistry, CBC levels and diabetes mellitus typing.

Index	Unstandardised coefficient		Standardised coefficient	t	*p*	95% CI	VIF
	B	standard error	Beta				
Age	0.012	0.001	0.577	10.861	0	0.010 ~ 0.014	2.68
ALT/AST	-0.047	0.019	-0.09	-2.482	0.013	-0.083 ~ -0.010	1.239
PA	0.001	0	0.153	3.126	0.002	0.000 ~ 0.001	2.266
HDL-C	-0.104	0.037	-0.095	-2.777	0.006	-0.177 ~ -0.031	1.112
TBIL	0.001	0.004	0.014	0.177	0.86	-0.007 ~ 0.009	5.575
IBIL	0.002	0.005	0.026	0.333	0.74	-0.008 ~ 0.012	5.77
ALP	0	0	0.023	0.64	0.522	-0.000 ~ 0.001	1.202
Crea	0	0	-0.007	-0.142	0.887	-0.001 ~ 0.001	2.408
RBP	0	0.001	0.005	0.091	0.927	-0.002 ~ 0.003	2.908
GFR	-0.001	0.001	-0.061	-0.834	0.404	-0.002 ~ 0.001	5.072
GLU	-0.002	0.002	-0.035	-0.771	0.441	-0.006 ~ 0.002	1.963
GSP	-0.024	0.02	-0.051	-1.162	0.246	-0.063 ~ 0.016	1.855
WBC	0.012	0.067	0.124	0.175	0.861	-0.120 ~ 0.144	475.526
NEU	-0.018	0.068	-0.178	-0.267	0.79	-0.151 ~ 0.115	423.5
LYM	-0.006	0.069	-0.012	-0.088	0.93	-0.141 ~ 0.129	16.63
MONO	-0.071	0.1	-0.046	-0.706	0.48	-0.267 ~ 0.125	3.966
EOS	-0.496	0.227	-0.219	-2.188	0.029	-0.941 ~ -0.052	9.53
EOS-R	0.034	0.016	0.205	2.091	0.037	0.002 ~ 0.065	9.123
BASO-R	-0.046	0.046	-0.037	-1	0.318	-0.136 ~ 0.044	1.308
RBC	0.036	0.021	0.07	1.693	0.091	-0.006 ~ 0.078	1.648
RDW-SD	0	0.003	-0.005	-0.142	0.887	-0.007 ~ 0.006	1.343
PLT	0.001	0	0.112	1.908	0.057	-0.000 ~ 0.001	3.275
PCT	-0.272	0.273	-0.057	-0.997	0.319	-0.807 ~ 0.263	3.059
Na+	-0.005	0.004	-0.051	-1.27	0.205	-0.011 ~ 0.002	1.555
CO2	0.004	0.004	0.055	1.119	0.263	-0.003 ~ 0.011	2.306
AG	0	0.004	0.002	0.039	0.969	-0.007 ~ 0.007	2.254
C-peptide	0.011	0.009	0.041	1.199	0	0.140~0.500	1.02

ALT/AST, alanine aminotransferase/aspartate aminotransferase; PA, prealbumin; HDL-C, high-density lipoprotein cholesterol; TBIL, total bilirubin; IBIL, indirect bilirubin; ALP, alkaline phosphatase; Crea, creatinine; RBP, retinol-binding protein; GFR, glomerular filtration rate; GLU, glucose; GSP, glycated serum proteins; WBC, white blood cell count; NEU, neutrophil count; LYM, lymphocyte count; MONO, monocyte count; EOS, eosinophil count; EOS-R, eosinophil ratio; BASO-R, basophil ratio; RBC, red blood cell count; RDW-SD, standard deviation in red blood cell distribution width; PLT, platelet count; PCT, platelet compact volume; Na^+^, sodium ions; CO_2,_ carbon dioxide; AG, anion gap.

### 3.3 The significant differences in ZnT8A, ICA , IA-2A and GADA between T1DM and T2DM groups

In addition, for independent samples, non-parametric test (Kruskal-Wallis) was performed for both T1DM and T2DM groups. Statistically significant differences were observed between the two groups for ZnT8A, ICA, IA-2A and GADA (p < 0.05), as indicated in the **Table 3**. However, no significant differences were noted for IAA (p > 0.05), which suggests that ZnT8A, ICA, IA-2A, and GADA may serve as the factors in the differentiation of T1DM from T2DM.

**Table 3:** Results of Kruskal-Wallis test.

Index	χ^2^	df	*p*-value	ϵ^2^
ZnT8A	5.9400	1	0.015	0.011401749
ICA	2.3100	1	0.046	0.004058273
IAA	0.0824	1	0.774	0.000158102
IA-2A	5.0400	1	0.025	0009671312
GADA	19.1000	1	< 0.001	0.036576088

ZnT8A, zinc transporter-8 autoantibodies; ICA, autoantibodies; IAA, insulin autoantibodies; IA-2A, islet antigen 2 autoantibodies; GADA, glutamic acid decarboxylase autoantibodies.

### 3.4 Age, PA, HDL-C, ICA, IA-2A, GADA, and C-peptide were potential predictors of TIDM prediction model

**Figure 1:**
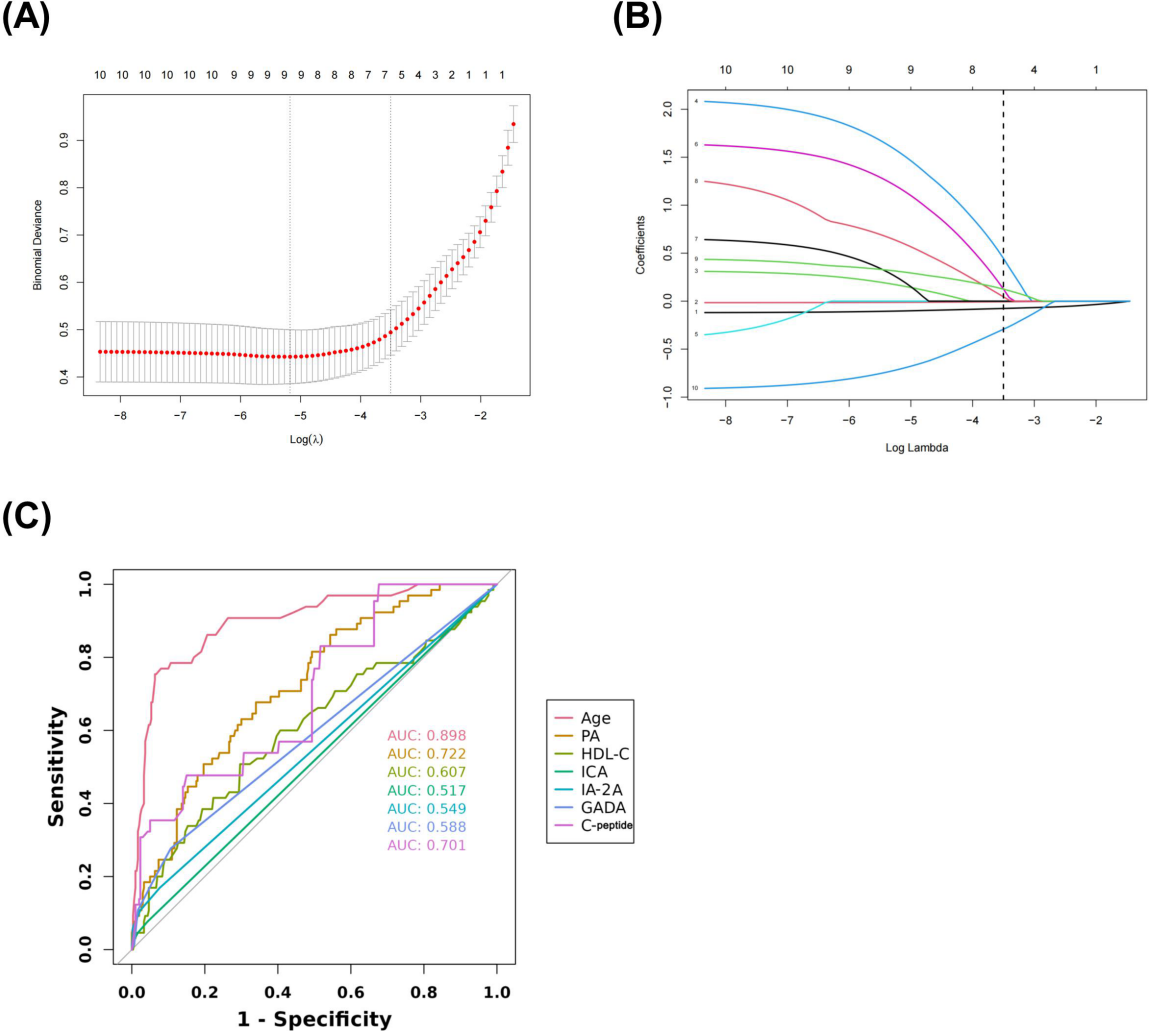
Lasso regression cross-validation Plot **(A)** Lasso regression coefficient path plot **(B)** Coefficients of Lasso regression analysis **(C)**.

The data included in this study were randomly divided into two groups in the ratio of 7:3 for the training and validation sets. Patients’ baseline data are provided in the **Supplementary Table 1**. These baseline characteristics provide a detailed overview of the study population and set the stage for further predictive research analysis. The candidate predictors i.e., age, PA, AST/ALT, HDL-C, C-peptide, and diabetes autoantibodies were included in the original model, which were then reduced to 7 potential predictors using LASSO regression analysis performed in the training cohort. The cross-validated error plot of the LASSO regression model is shown in the **Figure 1A**. The most regularized and parsimonious model, with a cross-validated error within one standard error of the minimum, included 7 variables. The coefficient profile is plotted in the **Figure 1B**. As depicted in the **Figure 1C**, the ROC analysis of the abovementioned variables yielded AUC values greater than 0.5. Further univariate and multivariate logistic analysis were performed as shown in the **Tables 4**, **5**.

**Table 4:** Results of univariate logistic regression.

Characteristic	N	Event N	OR	95% CI	*p*
Age	365	65	0.90	0.88, 0.92	<0.001
PA	365	65	0.99	0.98, 0.99	<0.001
HDL-C	365	65	3.07	1.38, 6.83	0.006
ICA	365	65	1.64	0.88, 3.06	0.037
IA-2A	365	65	1.97	1.29, 3.03	0.002
GADA	365	65	1.76	1.30, 2.38	<0.001
C-peptide	365	65	0.45	0.32, 0.61	<0.001

PA, prealbumin; HDL-C, high-density lipoprotein cholesterol; ICA, autoantibodies; IA-2A, islet antigen 2 autoantibodies; GADA, glutamic acid decarboxylase autoantibodies.

**Table 5:** Results of multivariate logistic regression for training cohort.

Characteristic	N	Event N	OR	95% CI	*p*
Age	365	65	0.89	0.86, 0.92	<0.001
PA	365	65	0.98	0.98, 0.99	<0.001
HDL-C	365	65	7.29	1.96, 27.08	0.003
ICA	365	65	4.79	1.58, 14.56	0.006
IA-2A	365	65	2.46	1.07, 5.65	0.034
GADA	365	65	1.43	0.87, 2.35	0.047
C-peptide	365	65	0.39	0.23, 0.66	<0.001

PA, prealbumin; HDL-C, high-density lipoprotein cholesterol; ICA, autoantibodies; IA-2A, islet antigen 2 autoantibodies; GADA, glutamic acid decarboxylase autoantibodies.

### 3.5 Construction and performance of nomogram

**Figure 2:**
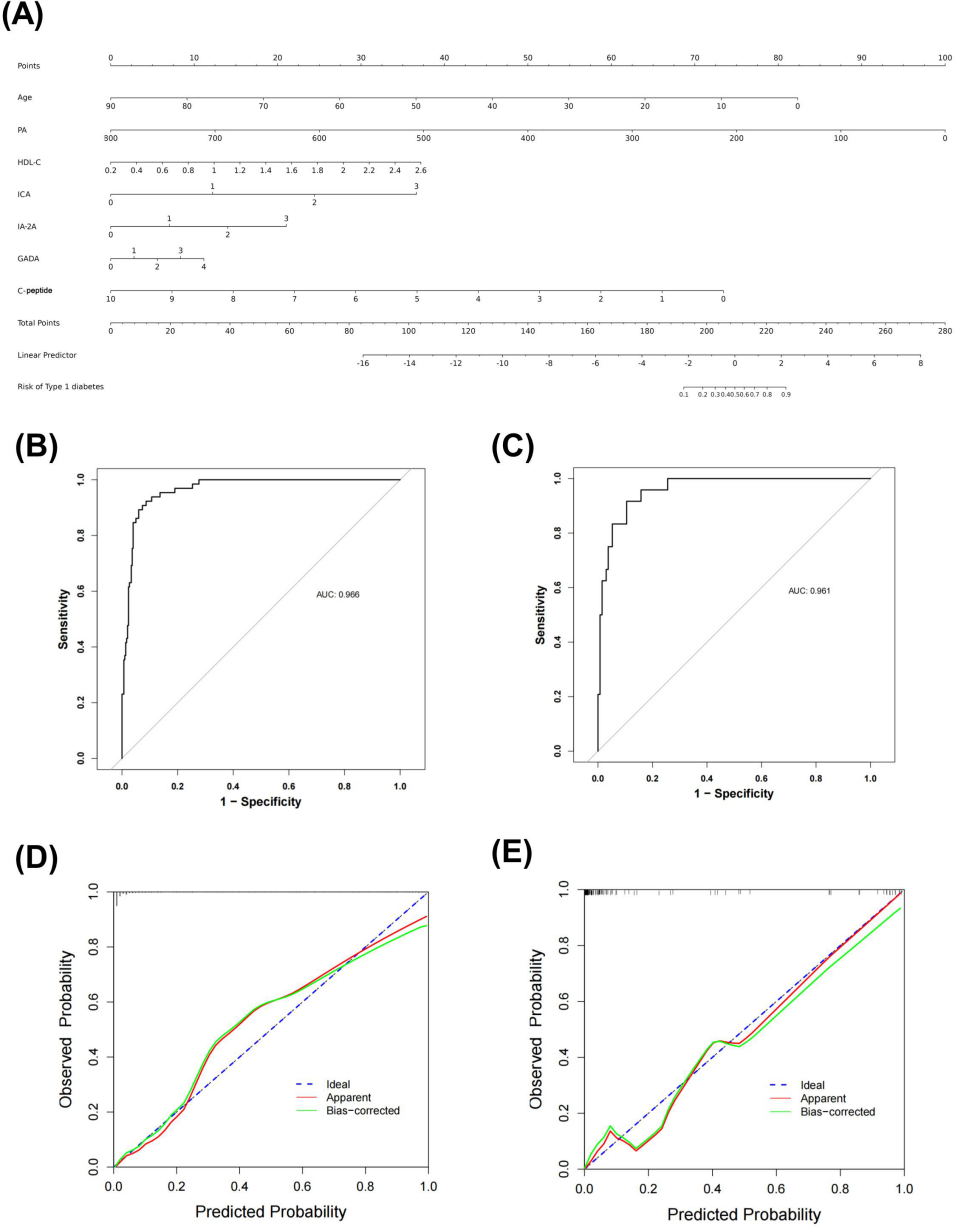
Nomogram predicting T1DM in patients with DM **(A)** ROC curve of the nomogram in training set **(B)** and validation set **(C)** Calibration curves of the nomogram prediction in training set **(D)** and validation set **(E)**.

The final logistic model included 7 independent predictors (age, PA, HDL-C, ICA, IA-2A, GADA, and C-peptide) and was developed as a simple-to-use nomogram to predict the probability of T1DM (**Figure 2A**). Each parameter was assigned an exact score. The sum of the scores in the graph is the total score, which corresponds to T1DM risk. In **Figure 1A**, a higher total score indicates a higher risk of T1DM. Plotting the ROC curve, in the training set, the Area Under Curve (AUC) was 0.966(**Figure 2B**). Meanwhile, in the validation set, the AUC also reached 0.961 (**Figure 2C**), indicating that the nomogram has good predictive ability. In addition, the calibration curves show that the nomogram is strongly calibrated in both the training set (**Figure 2D**) and the validation set (**Figure 2E**).

### 3.6 Practical applications of the nomogram

**Figure 3:**
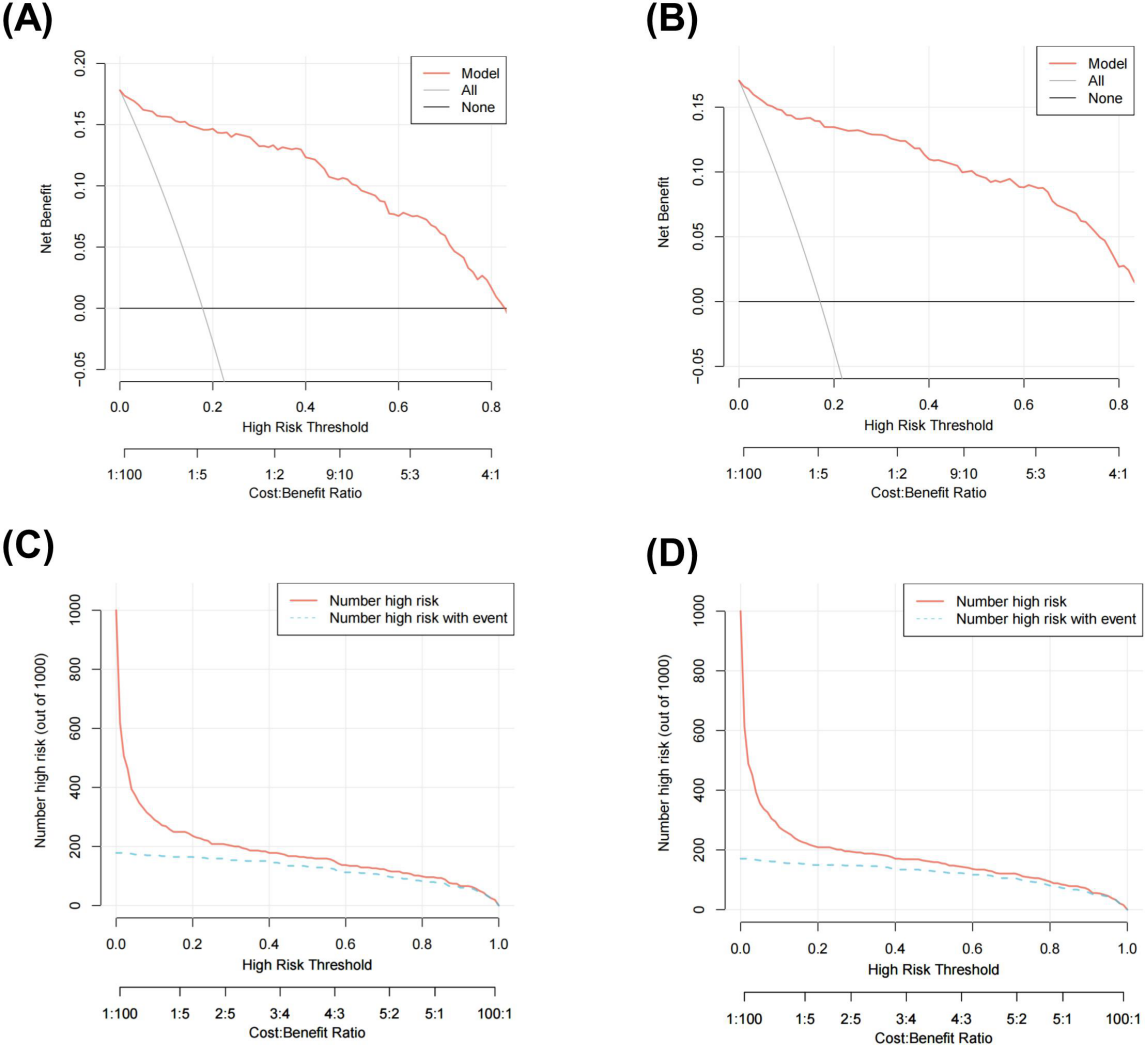
Decision curve analysis (DCA) of the nomogram in training set **(A)** and validation set **(B)**clinical impact curve (CIC) of the nomogram in training set **(C)** and validation set **(D)**.

The net benefit was examined using decision curve analysis (DCA) in order to further evaluate this predictive model. The results showed that the nomogram produced a net benefit relative to the treat-all-patients scenario or no-treatment scenario when the predictive probability of the nomogram for T1DM was less than 80% in both the training set (**Figure 3A**) and the validation set (**Figure 3B**), indicating that the nomogram had therapeutic value. To assess the nomogram’s clinical impact and illustrate its qualitative significance, the Clinical Impact Curve (CIC) was additionally plotted based on DCA result. The CIC demonstrated the nomogram’s strong predictive ability in both the training set (**Figure 3C**) and validation set (**Figure 3D**). **Figures 3C, D** illustrates the number of patients predicted to have T1DM and the number of patients who actually had T1DM at each risk threshold. When 20% is the risk threshold, the anticipated number of patients is closer to the actual number of patients.

### 3.7 Building a web application to view nomograms

**Figure 4:**
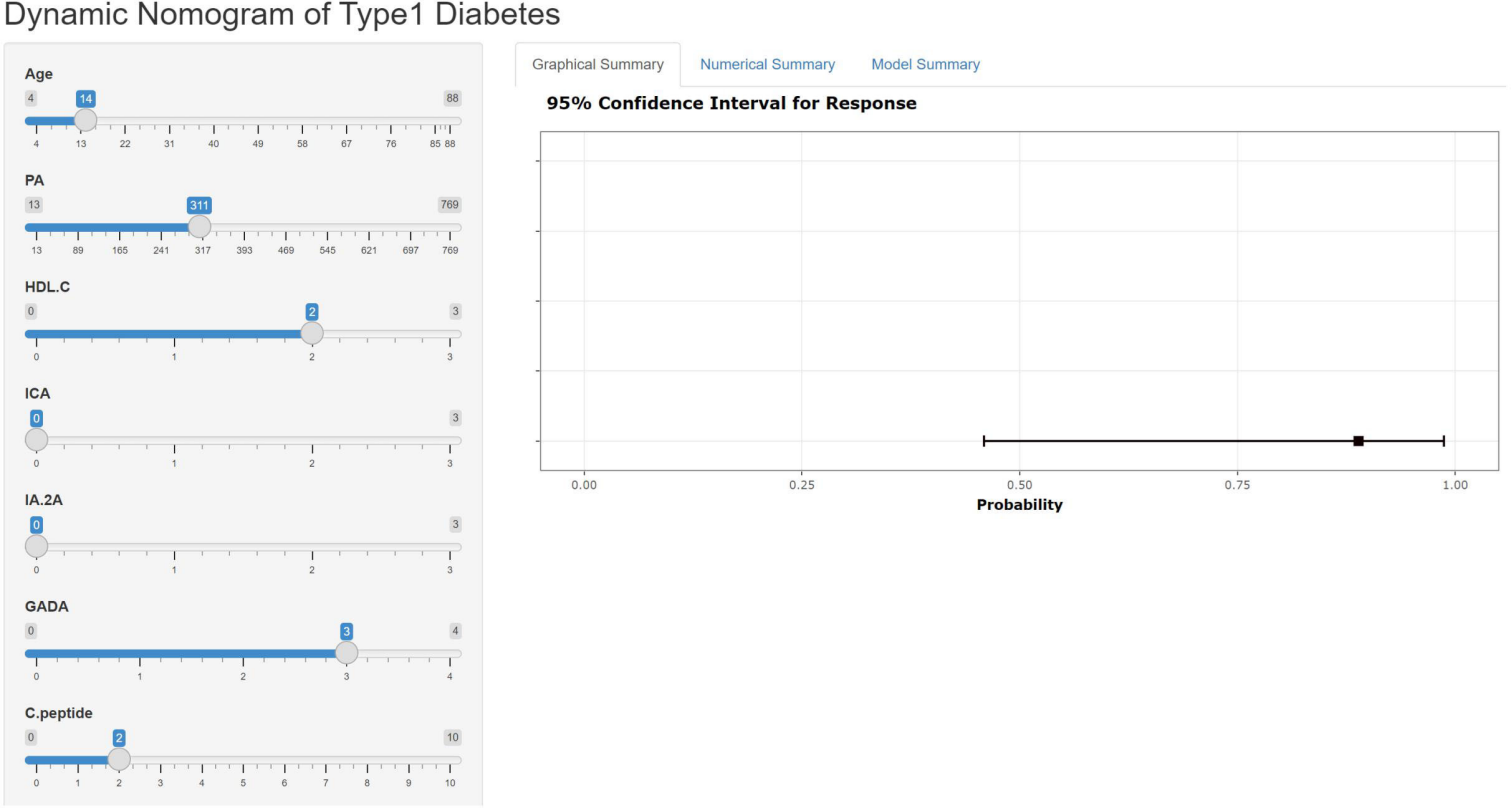
An example of T1DM prediction using the nomogram via a link.

The nomogram can be accessed by medical staff through our self-built web application at the given link (https://type1diabetesdiagnosis.shinyapps.io/dynnomapp/). The algorithm automatically calculates the probability of a patient having T1DM. The scoring system enables early differentiation of patients with T1DM and facilitates appropriate therapeutic measures. For example, when the patient is 14 years old, has a PA level of 311.00 mmol/l, an HDL-C level of 2.00 mmol/l, and a GADA level of +++, the probability of developing T1DM is 0.889 (**Figure 4**).

## 4 Discussion

Diabetes mellitus (DM) is a group of metabolic diseases characterized by hyperglycemia caused by defects in either insulin secretion or action, or both. Diabetes-related chronic hyperglycemia is linked to long-term damage, malfunction, and failure of several organs, including the heart, blood vessels, kidneys, eyes, and nerves (24). The healthcare expenditures and access to treatment are unequal between developed and developing countries, nevertheless, both bear a huge financial burden (25, 26). Early and accurate identification of DM is important for determining treatment options, improving outcomes and reducing the economic burden. However, there are limitations in classifying T1DM and T2DM based on the clinical data, laboratory metabolic testing, and diabetes autoantibody testing (8, 9, 14–17). Additionally, the value of routine laboratory tests for typing is unclear. In this study, we constructed a quantifiable and simple nomogram for predicting T1DM, which can help clinicians to differentiate between T1DM and T2DM, and which contains one clinical parameter (age), two routine laboratory tests (PA, HDL-C), one islet β-cells function assessment test (C-peptide) and three diabetic autoantibody tests (GADA, ICA, IA-2A).

There is a clear distinction between the age of onset of T1DM and T2DM. The onset of T1DM is usually between 5-7 years of age and adolescence, but can occur at any age (7), while the onset of T2DM occurs after puberty. In the present study, the mean age of patients in the T1DM group was 28.7 years, which was significantly lower than that of the T2DM group, which was 58.5 years (p < 0.05). Moreover, in the Logistic regression model, the regression coefficient of age was 0.012 (p < 0.05), indicating that age is a significant influence on DM typing.

Prealbumin (PA) is a negative acute phase response protein and non-specific host defense substance, mainly synthesized by the liver, with a half-life of about 2 days, which makes it more sensitive compared to albumin, which has a half-life of 20 days, and has been used in clinical practice mainly to assess hepatic impairment and malnutrition (27). In recent years, it has been discovered that PA contributes to autoimmune diseases. One study (28) has shown that PA levels were negatively correlated with the degree of autoimmunity, which is consistent with the negative correlation between PA and T1DM typing in this study. In the present study, PA levels in the T2DM group were significantly higher than those in the T1DM group and were positively correlated with T2DM, which confirms that patients with T2DM are more prone to cardiovascular disease (29). Nicoletta Dozio et al. (30) also showed that PA levels vary at different stages of T1DM disease course, with lower levels in patients with longer disease duration, and this study is expected to play a role in evaluating the stage of disease and prognosis of patients at the time of initial diagnosis of T1DM.

High-density lipoprotein cholesterol (HDL-C) has anti-atherosclerotic and antioxidant properties and prevents oxidized LDL (ox-LDL) interceded endothelial dysfunction (31). Characteristic dyslipidemia usually precedes the diagnosis of T2DM, such as reduced HDL-C levels, suggesting that reduced HDL-C promotes the onset and progression of T2DM and diabetic vascular complications (32). Indeed, it has been found that there is a bidirectional association between HDL-C and T2DM, whereby hyperglycemia and hyperinsulinemia occurring in T2DM may lead to reduced HDL-C levels and deterioration of HDL function through various alterations in the HDL particles proteome and lipidome (33).Thus, via altering insulin secretion, peripheral insulin sensitivity, non-insulin-dependent glucose uptake, and adipose tissue metabolic activation, HDL-C may also have an impact on glucose homeostasis (34). In the present study, the mean HDL-C values in the T2DM group were lower than those in the T1DM group, and there was a negative correlation between the HDL-C values and the T2DM phenotype, which is consistent with the findings mentioned above.

As described in 1967 (35), C-peptide is a 31-amino acid peptide, facilitating the correct folding of insulin and formation of its disulfide bridges. Proinsulin is cleaved into insulin and C-peptide. These two proteins are stored in the secretory granules of the pancreatic β-cells and eventually released together in equimolar amounts. C-peptide has negligible extraction by the liver and constant peripheral clearance. Its half-life is longer than insulin (20–30 vs. 3–5 min) (36). Therefore, the physiology of C-peptide makes it appropriate for assessing insulin secretion. Absolute insulin deficiency is a key feature of Type 1 diabetes, and C-peptide levels taken within the first few years of diagnosis may be useful in confirming Type 1 diabetes if results are low (37). As such, C-peptide has been a valuable tool in elucidating the pathophysiology of T1DM and T2DM. In the present study, the mean C-peptide values in the T2DM group were higher than those in the T1DM group, which is consistent with the above-mentioned findings.

Autoimmunity and cellular immunity in T1DM patients contribute to the onset and progression of the disease. Pertinently, some scholars have proposed that diabetic autoantibody detection in patients’ serum can be an effective diagnostic method for typing of diabetic patients, and currently the clinical use of antibodies including GADA, IAA, IA-2A, ZnT8A, ICA (12). Among diabetic autoantibodies, the highest positive rate belongs to the Glutamic acid decarboxylase (GAD) antibody. GAD is a key enzyme in the synthesis of inhibitory neurotransmitter γ-aminobutyric acid, and the available data confirm that its level can be elevated several years or even more than 10 years prior to the onset of T1DM. Moreover, it has the characteristics of high sensitivity and specificity, and is considered to be a specific marker for immune destruction of pancreatic islet β-cells in T1DM patients (38). GADA is the earliest antibody to GAD, and some scholars have found that a single positive GADA has a predictive value for insulin β-cell function (39). IAA was discovered in 1983 in T1DM patients who had not used exogenous insulin (40). Subsequent studies have shown that anti-insulin antibodies are present prior to the onset of T1DM (41), and were negatively correlated with age at onset of T1DM (42). ICA is a cytoplasmic antibody to pancreatic islet β-cells, which can cause an immune response upon binding to islet cell surface antigen, resulting in cytotoxic effects on islet cell cytoplasmic components, leading to cell lysis, death, and ultimately DM. Also, ICA is the first diabetic autoantibody found to be associated with the development of T1DM disease (43). According to earlier research, ICA is present in approximately 70% of T1DM patients, but for a short period of time i.e., appearing only in the early stages of T1DM (15). Positive ICA is now considered to be indicative of autoimmune damage to pancreatic β-cells and is highly predictive of T1DM when it is persistently positive or at high levels.

The results of the present study demonstrated that the difference in the positive rates of ZnT8A, ICA, IA-2A and GADA between patients in the T1DM and the T2DM group was statistically significant (p < 0.05), suggesting that the pancreatic β-cells had undergone a strong autoimmune reaction, which had caused impaired insulin secretion from the β-cells, leading towards pancreatic β-cell failure, which was in line with the main characteristics of T1DM. Between the two groups, there was no statistically significant difference in the positive rates of IAA (p > 0.05).

In this study, the following seven predictors were selected: age, PA, HDL-C, ICA, IA-2A, GADA, and C-peptide. A multivariate predictive model, nomogram, was established with excellent efficacy, and it could distinguish T1DM well, with an AUC of 0.966 and 0.961 in the training set and validation set, respectively. According to the calibration curves, the nomogram has a strong calibration. Moreover, it can serve as a useful tool for clinical applications and lower the cost and burden of disease, according to subsequent DCA and CIC assessments. Finally, we have built a web-based computational tool that may facilitate doctors’ by providing a platform for convenient and enhanced application of the nomogram. A previous study (44) built a similar predictive model that included age, body mass index, FPG, and TC to focus on the risk of developing T2DM in hypertensive patients. Another study (45) developed and validated a personalized prediction nomogram for non-obese adults with 5-year T2DM risk, including age, GGT, TG, FPG, HbA1c, and fatty liver. In our study, we introduced a novel predictive model integrating autoantibody profiles with clinical and laboratory data, and the differential prediction of T1DM and T2DM was carried out, which is a supplement to the former and the field. Unlike existing models that often rely on single diagnostic criteria or limited parameters, our approach aims to significantly enhance classification accuracy by considering a comprehensive set of predictors.

However, there are still some limitations in this study. First, our participants were all patients from the same hospital, which may make the results not applicable to other countries and regions. Second, we excluded patients with incomplete data, leading to potential selection bias inherent in our participant recruitment process. Future research efforts should prioritize addressing these limitations through larger, multicenter studies involving diverse patient populations and rigorous validation protocols. Such endeavors would enhance the generalizability and reliability of predictive models for classifying diabetes mellitus types. By expanding the scope of research beyond single-center studies and incorporating broader patient demographics, we can strengthen the evidence base supporting clinical decision-making in diabetes care.

Despite these limitations, our findings remain a supporting tool for clinical decision making. Our study demonstrates that the predictive nomogram integrating specific autoantibodies and laboratory indices can significantly improve the accuracy of diabetes mellitus classification. It provides quantitative predictions based on individual patient data, aiding clinicians in making informed decisions about treatment, monitoring requirements, and patient education efforts. However, implementing the nomogram in clinical settings may present challenges. Healthcare providers must ensure access to the necessary laboratory tests and interpret the nomogram’s results within the context of each patient’s clinical presentation and medical history. Training and education for healthcare providers on the use and interpretation of the nomogram are essential to optimize its utility and minimize the risk of misclassification or misinterpretation of results.

In conclusion, the application of individual clinical data, routine laboratory indicators or diabetes autoantibodies in the diagnosis and treatment of DM is relatively limited, and it is necessary to comprehensively consider age, PA, HDL-C, ICA, IA-2A, GADA, and C-peptide. Conclusively, the nomogram that is created based on these variables may provide useful differentiation between T1DM and T2DM, and the assessment of changes through the course of DM, which can provide a scientific guide to clinicians for diabetes prevention and treatment.

## Data Availability

The original contributions presented in the study are included in the article/**Supplementary Material**. Further inquiries can be directed to the corresponding author.
