# Supplementary material for: The application of predictive value of diabetes autoantibody profile combined with clinical data and routine laboratory indexes in the classification of diabetes mellitus
Source: Front Endocrinol (Lausanne). 2024 Aug 22;15:1349117. doi: 10.3389/fendo.2024.1349117 (PMC11377899; doi:10.3389/fendo.2024.1349117)
Supplement: Supplementary file 1 [file Table1.docx]

Supplementary Table 1. Patient demographics and baseline characteristics

|  | Cohort | | *p* |
| --- | --- | --- | --- |
|  | Training Cohort, N = 365 | Internal Test Cohort, N = 157 |  |
| Age |  |  | 0.330 |
| Mean ± SD | 53 ± 18 | 55 ± 17 |  |
| PA |  |  | 0.934 |
| Mean ± SD | 215 ± 73 | 216 ± 87 |  |
| AST/ALT |  |  | 0.744 |
| Mean ± SD | 1.07 ± 0.72 | 1.09 ± 0.71 |  |
| HDL-C |  |  | 0.173 |
| Mean ± SD | 1.11 ± 0.33 | 1.16 ± 0.38 |  |
| ZnT8A |  |  | 0.858 |
| Median (IQR) | 0.00 (0.00, 0.00) | 0.00 (0.00, 0.00) |  |
| ICA |  |  | 0.103 |
| Median (IQR) | 0.0000 (0.0000, 0.0000) | 0.0000 (0.0000, 0.0000) |  |
| IAA |  |  | 0.128 |
| Median (IQR) | 0.00 (0.00, 0.00) | 0.00 (0.00, 0.00) |  |
| IA-2A |  |  | 0.620 |
| Median (IQR) | 0.00 (0.00, 0.00) | 0.00 (0.00, 0.00) |  |
| GADA |  |  | 0.742 |
| Mean ± SD | 0.26 ± 0.73 | 0.24 ± 0.68 |  |
| C-peptide |  |  | 0.060 |
| Mean ± SD | 1.98 ± 1.49 | 1.74 ± 1.30 |  |

PA: prealbumin; ALT/AST: alanine aminotransferase/aspartate aminotransferase; HDL-C: high-density lipoprotein cholesterol; ZnT8A: zinc transporter-8 autoantibodies; ICA: autoantibodies; IAA: insulin autoantibodies; IA-2A: islet antigen 2 autoantibodies; GADA: glutamic acid decarboxylase autoantibodies.
